# In silico and in vitro models reveal the molecular mechanisms of hypocontractility caused by *TPM1* M8R

**DOI:** 10.3389/fphys.2024.1452509

**Published:** 2024-08-30

**Authors:** Jenette G. Creso, Ilhan Gokhan, Michael J. Rynkiewicz, William Lehman, Jeffrey R. Moore, Stuart G. Campbell

**Affiliations:** ^1^ Department of Biomedical Engineering, Yale University, New Haven, CT, United States; ^2^ Department of Pharmacology, Physiology and Biophysics, Boston University Chobanian and Avedisian School of Medicine, Boston, MA, United States; ^3^ Department of Biological Sciences, University of Massachusetts–Lowell, Lowell, MA, United States; ^4^ Department of Cellular and Molecular Physiology, Yale School of Medicine, New Haven, CT, United States

**Keywords:** dilated cardiomyopathy, tropomyosin, computational modeling, engineered heart tissue, disease modeling

## Abstract

Dilated cardiomyopathy (DCM) is an inherited disorder often leading to severe heart failure. Linkage studies in affected families have revealed hundreds of different mutations that can cause DCM, with most occurring in genes associated with the cardiac sarcomere. We have developed an investigational pipeline for discovering mechanistic genotype-phenotype relationships in DCM and here apply it to the DCM-linked tropomyosin mutation *TPM1* M8R. Atomistic simulations predict that M8R increases flexibility of the tropomyosin chain and enhances affinity for the blocked or inactive state of tropomyosin on actin. Applying these molecular effects to a Markov model of the cardiac thin filament reproduced the shifts in Ca^2+^sensitivity, maximum force, and a qualitative drop in cooperativity that were observed in an *in vitro* system containing *TPM1* M8R. The model was then used to simulate the impact of M8R expression on twitch contractions of intact cardiac muscle, predicting that M8R would reduce peak force and duration of contraction in a dose-dependent manner. To evaluate this prediction, *TPM1* M8R was expressed via adenovirus in human engineered heart tissues and isometric twitch force was observed. The mutant tissues manifested depressed contractility and twitch duration that agreed in detail with model predictions. Additional exploratory simulations suggest that M8R-mediated alterations in tropomyosin-actin interactions contribute more potently than tropomyosin chain stiffness to cardiac twitch dysfunction, and presumably to the ultimate manifestation of DCM. This study is an example of the growing potential for successful *in silico* prediction of mutation pathogenicity for inherited cardiac muscle disorders.

## 1 Introduction

Ca^2+^ regulation of the cardiac sarcomere is achieved by the regulatory proteins tropomyosin (Tpm, encoded by the gene *TPM1*) and troponin which lie on actin’s surface (for a full review see Gordon et al. (Gordon et al., 2000)). Tpm is a dimeric coiled-coil protein that polymerizes in a head-to-tail overlap fashion along the length of the actin filament. Structural studies continue to enhance our understanding of the detailed mechanisms of thin filament regulation (Yamada et al., 2020; Risi et al., 2024; Risi et al., 2023; Risi et al., 2021). Ca^2+^ binding to troponin C (TnC) initiates a sequence of conformational changes that results in azimuthal shifting of Tpm on actin’s surface. Azimuthal shifting defines three regulatory states of Tpm: blocked (B) in which Tpm is blocking the myosin binding sites on actin; closed (C) in which troponin has conformationally switched to allow Tpm to partially shift off of the binding sites; and open (M) in which binding of myosin causes further shifting of tropomyosin (McKillop and Geeves, 1993; Vibert et al., 1997). Given their potential to affect strength and duration of cardiac contraction, it is not surprising that mutations in Tpm have been linked to genetic diseases such as dilated cardiomyopathy (DCM) (Mirza et al., 2007; Lakdawala et al., 2009; Chang and Potter, 2005; Olson et al., 2001; Lynn et al., 2017; Daehmlow et al., 2002; Redwood and Robinson, 2013). DCM is characterized by a thinning of the left ventricular wall and increased chamber size, coupled with systolic dysfunction which often results in morbidity and mortality related to heart failure and arrhythmias (Dilated cardiomyopathy, 2019).

Stability of tropomyosin binding to actin is influenced considerably by the end-to-end overlap between adjacent protein dimers (Palm et al., 2003). In addition, this overlap contributes to the ability of a single tropomyosin to cooperatively influence its nearest neighbors, while at the same time acting as a receptor for the tail domain of the troponin complex (Moore et al., 2016). As such, mutations that exist in the overlap region of Tpm are expected to have severe effects if they disrupt the normal affinity of Tpm for its regulatory positions on actin or the effective stiffness of the Tpm chain. One such DCM-linked mutation results in the replacement of the methionine at residue 8 with an arginine (M8R) in Tpm (Racca et al., 2020; Matyushenko et al., 2019). This mutation has been found in two separate screens of DCM patients (Pugh et al., 2014; Lakdawala et al., 2010) and a search for *TPM1* M8R in the gnomAD database did not produce any results, indicating that this variant is not commonly seen in the general population. Previous work explored the structural effects of M8R using molecular dynamics (MD) simulations, *in vitro* motility assays (IVMA), and actin co-sedimentation (Racca et al., 2020). It was seen that introducing the mutant tropomyosin resulted in decreased Ca^2+^ sensitivity and decreased Ca^2+^-activated cooperativity in the IVMA. The hypothesis, influenced heavily by the MD simulations, was that M8R caused these changes through weakened tropomyosin-tropomyosin interactions, weakened tropomyosin-actin interactions, and a shift in the equilibrium position of tropomyosin towards the B-state on actin’s surface.

In this study, we extended these results using both *in silico* modeling and tissue engineering approaches to provide mechanistic insight for how the mutation-induced molecular effects influence more physiologically relevant cardiac tissue function. By inputting the structural and mechanical changes predicted by MD for M8R into a thin filament activation model, we were able to recapitulate measured steady-state behavior from the IVMA. Applying the same parameter changes to simulations of isometric twitch predicted contractile dysfunction proportional to the level of M8R expression. Using adenovirus transduced engineered heart tissue expressing *TPM1* M8R, we were able to corroborate the mechanistic insights fed into the model and analyze the accuracy of its predictions. This study demonstrates the potential for a multiscale computational approach to predict genotype-phenotype relationships for sarcomeric variants.

## 2 Methods

### 2.1 Computer simulations

Simulations for both steady-state and isometric twitches were performed using the thin filament model published previously in Creso and Campbell (Creso and Campbell, 2021) (see Table 1 for parameter sets). This Markov model simulates the behavior of 26 regulatory units coupled in series to form a virtual thin filament. Each regulatory unit behaves according to a 24-state model that describes the ensemble status of myosin, tropomyosin, and key domains of troponin C and troponin I. The simulation produces a prediction of force over a set time course with either a steady-state Ca^2+^ concentration or a transient. The model can be driven by any arbitrary input Ca^2+^ waveform. Force was calculated as the number of regulatory units in a myosin-bound state, corresponding to tropomyosin in the M-state.

**TABLE 1:** Parameter set used in simulations. (Set 1) steady-state model parameters; (Set 2) kinetic model parameters. Parameters are defined in Creso and Campbell (Creso and Campbell, 2021).

Parameter	Set 1	Set 2
$k_{Ca}^{+}\;\left({\mu M^{-1}s}^{-1}\right)$	350	310
$k_{Ca}^{-}\left(s^{-1}\right)$	1000	1800
$k_{SP}^{+}\left(s^{-1}\right)$	180	225
$k_{SP}^{-}\left(s^{-1}\right)$	292	350
$k_{IP}^{+}\left(s^{-1}\right)$	700	1900
$k_{IP}^{-}\left(s^{-1}\right)$	225	225
$k_{MD}^{+}\left(s^{-1}\right)$	590	1650
$k_{MD}^{-}\left(s^{-1}\right)$	225	225
$k_{ref}^{BC}\left(s^{-1}\right)$	675	875
$K_{BC}$	1.39−2.3	1.57−2
$f_{XY}\left(s^{-1}\right)$	225	25
δ	0.48	0.4
λ	0.008	0.0001
η	9	18
μ	9	18
$\gamma \left(mol^{-1}kJ\right)$	35 70	25 50

The number of actin-myosin crossbridges (corresponding to M-state tropomyosin) at steady-state for various Ca^2+^ concentrations were obtained by simulating a 10 s interval and averaging model output over a window of the final 2.5 s of each simulation. Steady-state force values at different Ca^2+^ concentrations were used to produce force-pCa plots which were fit using the Hill equation. To fit our model to experimental IVMA data, we assumed approximate proportionality between actin sliding velocity and the average number of attached actin-myosin crossbridges predicted by the model at steady-state for given pCa, as we have done previously (Sewanan et al., 2021). This approximation seems reasonable for the assays reported by Racca *et al* (Racca et al., 2020). but plainly does not hold for all possible conditions (Uyeda et al., 1990).

Twitch data was recorded by allowing the system to reach steady-state at diastolic Ca^2+^concentration of 0.1 μM. The Ca^2+^ concentration was then allowed to produce a transient by increasing up to 1 μM based on data from Stull et al. (Stull et al., 2002) (Figure 2A).

The model was scripted, and post-processing was conducted in MATLAB, while the Markov chain-Monte Carlo algorithm was implemented in CUDA C++ for parallel processing. Simulations were executed on an Nvidia GeForce RTX 2080Ti graphics processing card.

To ensure convergence of the stochastic model, the simulation time course was repeated 1920 times on the GPU and the average force at each time step was calculated. To reduce noise, twitch simulations were run 10 times and averaged to plot and calculate twitch properties (peak force, time to peak force, time to 50% relaxation, and normalized force-time integral).

Experimental data from Racca et al. identified two mechanistic perturbations related to M8R that we represented as mutation-based changes to the parameters γ and K_BC_ within the Markov model (Racca et al., 2020). γ encodes the effective chain stiffness of tropomyosin which scales the transition rates of tropomyosin by coupling its movement to the status of its nearest neighbors. Decreasing this parameter reduces the influence of neighboring tropomyosins and reduces cooperativity of the system. K_BC_ is the ratio of the tropomyosin B→C transition rate to the C→B transition rate. Reducing this parameter biases tropomyosin toward the B-state.

### 2.2 Tropomyosin vectors

WT *TPM1* vectors were purchased from OriGene. Mutagenesis to produce the M8R point mutation was performed using QuikChange II Site-Directed Mutagenesis Kit (Agilent Cat. No. 200523). The LFEAP method (Zeng et al., 2018) was used to introduce a FLAG tag onto the C-terminus of both WT and mutant tropomyosin vectors. All plasmids were sequenced-verified before use.

### 2.3 Production of recombinant adenovirus

Production of adenoviral vectors was done using the AdEasy XL Adenoviral System from Agilent Technologies (Cat. No. 240010) and followed their standard protocol. The gene was cloned into the pShuttle-IRES-hrGFP-1 vector from Agilent Technologies (Cat. No. 240081). Viral titers were measured using the AdEasy Viral Titer Kit from Agilent Technologies (Cat. No. 972500).

### 2.4 Tissue scaffolds

Engineered heart tissue was created using methods as described earlier in Schwan et al. (Schwan et al., 2016). with modifications. Briefly, isolated blocks of porcine left ventricle are flash frozen and then cryosectioned into 150 μm slices. These slices are thawed and laser cut into rectangular strips. The strips are clipped into a custom culture frame and decellularized by incubating in lysis buffer (10 mM Tris +5.4 mM EDTA, pH 7.4) for 2 hours at room temperature followed by a second incubation in a 0.5% SDS solution, gently agitating at room temperature for 45 min. Decellularized scaffolds are washed with DPBS and then incubated overnight in DMEM +10% FBS +2% penicillin-streptomycin (P/S) before seeding.

### 2.5 Cell culture

A control stem cell line (GM23338, Coriell Institute) was maintained in mTeSR media (STEMCELL Technologies). A standard differentiation protocol was used (Lian et al., 2013). Briefly: 15 μM chiron (CHIR99021, STEMCELL Technologies) was added on day 0 for 24 h and 5 μM IWP-4 (STEMCELL Technologies) was added on day 3 for 48 h. The cells were cultured in RMPI supplemented with B27, minus insulin until day 9 when the supplement was changed to B27 with insulin. A 4-day 4 mM lactate selection from day 12 was used for purification. Cells were seeded on day 18–20 of their differentiation process.

To seed, decellularized scaffolds were placed face-down in a custom-made PDMS bath. Cardiomyocytes were mixed with adult human cardiac fibroblasts (306-05A, PromoCell) at a ratio of 9:1 and seeded at 1 million cells per scaffold in seeding media (high glucose DMEM +10% FBS +1% sodium pyruvate +1% L-glutamine +1% non-essential amino acids +1% P/S) and incubated overnight at 37°C. The following day, tissues were flipped face-up into a 12-well plate and cultured with RPMI + B27. Media was changed every 2 days thereafter.

All tissues within the same batch were made from 1) scaffolds made at the same time from the same frozen block of porcine left ventricle, 2) the same differentiation batch of iPSC-CMs and 3) fibroblasts from the same culture batch. After seeding, tissues were randomly assigned to be transduced with WT *TPM1*, WT *TPM1* + FLAG, or *TPM1* M8R + FLAG.

### 2.6 Gene transfer

Tissues were cultured for an initial 1 week after flipping. On day 8, the tissues were infected with the viral stocks at an MOI of 250 in RPMI + B27 for 48 h. Tissues were cultured for an additional 5 days post-infection. After a cumulative 2 weeks of culture between pre- and post-adenoviral transduction, the tissues were mechanically tested on day 15 (Figure 3B).

### 2.7 Protein expression

For Western blot, presence of tropomyosin was tested using rabbit anti-tropomyosin (Cell Signaling Technologies, 1:1000) and FLAG using Monoclonal M2 mouse anti-FLAG (Sigma, 1:1000). Goat anti-Mouse 800CW and Donkey anti-rabbit 680RD IRDye secondary antibodies were purchased from LiCor (1:15,000). Controls were pig left ventricle to express only native tropomyosin and HEK cells (AD-293, Agilent) transduced with *TPM1* + FLAG at MOI = 5 to express only FLAG-tagged *TPM1*. Tissues and cells were lysed in RIPA buffer SDS-PAGE was run on 12% Mini-PROTEAN^®^ TGX™ Precast Protein Gels (BIO RAD) at 100V for 2 h 15 min. Gels were run as technical replicates. Analysis of bands was done using ImageJ. For raw blots, see Supplementary Figure S1.

Silver staining of protein gels to analyze myosin heavy chain isoform was performed largely as previously described (Chung et al., 2014). Briefly, frozen EHTs were resuspended in a high-potassium lysis buffer containing (in mM): KCl (190), KH_2_PO_4_ (100), K_2_HPO_4_ (50), EDTA (10), Na_4_O_7_P_2_ (10 mM), beta-mercaptoethanol (4), pH 6.5, with 5% Triton X-100, supplemented with protease and phosphatase inhibitors. After homogenization at 4°C for 1 h and clarification of the lysate at 13,000 rcf for 30 min, sample concentration was calculated using Bradford assay (Bio-Rad). Approximately 1 μg protein was run on a 7% polyacrylamide gel containing 10% SDS for 16–18 h at 4 mA constant current in a running buffer containing 50 mM Tris, 75 mM glycine and 0.05% SDS (inner buffer concentration: 5x running buffer with 20 mM beta-mercaptoethanol). Gels were silver stained (Bio-Rad Silver Stain Plus) according to the manufacturer’s instructions and scanned using a desktop scanner. Mouse ventricle and porcine left ventricle were used as standards for alpha- and beta-myosin heavy chain, respectively. The raw blot is shown in Supplementary Figure S2.

### 2.8 Mechanical testing

Methods for performing mechanical testing of engineered heart tissue have been described earlier in Schwan et al. (Schwan et al., 2016) In short, the tissue is loaded into a custom testing apparatus and immersed in a temperature-controlled bath containing Tyrode’s solution (in mM: 140 NaCl, 5.4 KCl, 1.8 CaCl_2_, 1 MgCl_2_, 25 HEPES, and 10 glucose; pH 7.3). Throughout the experiment, the bath is continuously superfused with Tyrode’s using computer-controlled syringe pumps. One end of the tissue is attached to a force transducer and the other onto a linear actuator. A custom MATLAB script was used to record the raw mechanical data of twitches during isometric stretch and electrical pacing.

Three total data collections were performed (N = 3). Collections 1 and two contained tissues from all three groups and collection three contained only WT *TPM1* + FLAG and *TPM1* M8R + FLAG. Sample size (n) for each data collection are as follows: WT *TPM1* n = 5,7 for a total of 12; WT *TPM1* + FLAG n = 5,10,7 for a total of 22; *TPM1* M8R + FLAG n = 5,10,8 for a total of 23. All data were collected prior to any statistical analysis.

### 2.9 Statistical analysis

Analysis of functional data for WT + FLAG and M8R + FLAG was performed using Student’s t test within Prism 9. Significance was determined if the returned *p* values were <0.05.

Previous studies have shown that the addition of a C-terminal FLAG tag should have minimal impact on function (Michele et al., 2002; Michele et al., 1999). However, we performed a *post hoc* bootstrap analysis (Hinkley, 1988) to compare the WT and WT + FLAG data. This analysis was run in MATLAB (code adapted from https://courses.washington.edu/matlab1/Bootstrap_examples.html) with 10,000 repeated samplings. This showed no significant difference between WT and WT + FLAG (Supplementary Figure S3).

## 3 Results

### 3.1 Steady-state simulations

**FIGURE 1:**
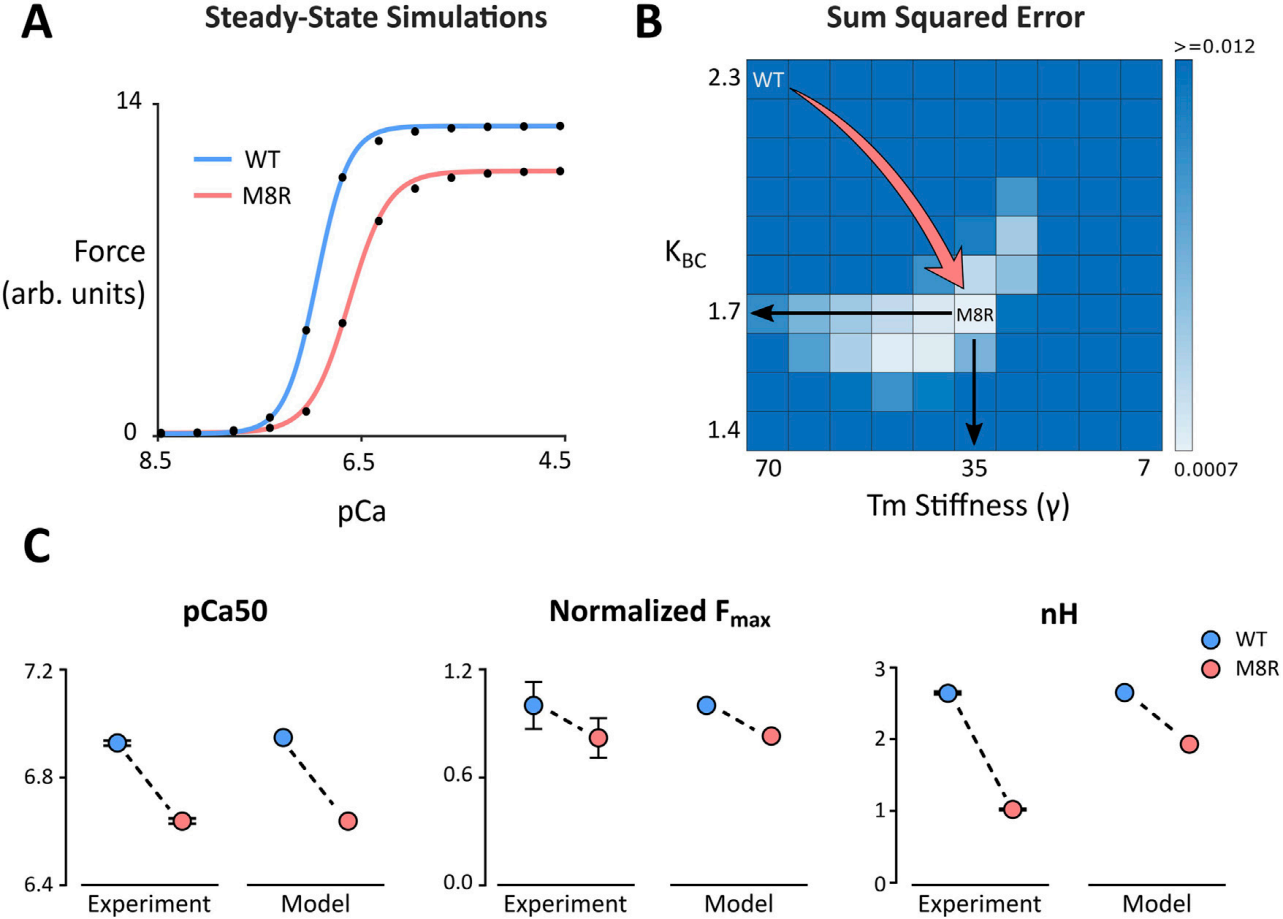
**(A)** Steady-state simulations comparing WT and M8R. Symbols show model output and solid lines show the fit of the Hill Equation; **(B)** Grid search heat map used to identify values for γ and K_BC_ that produce a sum of squared errors minimum during comparison of model output and IVMA data; **(C)** Change in pCa_50_, normalized maximum force, and Hill coefficient (n_H_) from WT to M8R compared between IVMA experimental data and computer simulations.

Our first objective was to find model parameters for the 24-state Markov model of Creso and Campbell (Creso and Campbell, 2021) that would reproduce effects of *TPM1* M8R as observed in the *in vitro* motility assay (IVMA) data of Racca et al. (Racca et al., 2020). A parameter set was first found that reproduced both the pCa_50_ and Hill coefficient (n_H_) of actin filaments decorated with wildtype (WT) tropomyosin (Figure 1A; Table 1). Specific model parameters that could rationally be adjusted in order to reproduce the effects of the tropomyosin M8R mutation were then selected based on our previous MD simulations (Racca et al., 2020). Although MD-predicted molecular changes were associated with specific regions of the tropomyosin molecule, these were represented as global changes to tropomyosin behavior in the Markov model owing to its coarse-grained formulation.

MD predicts that the location of the mutation within the end-to-end overlap region of tropomyosin creates a disruption that results in a decrease in persistence length or rigidity of the overlap structure. This change was incorporated into our Markov model as a decrease in the effective chain stiffness of tropomyosin with the parameter gamma (γ), which ultimately reflects the extent to which a single tropomyosin influences the state transitions of its nearest neighbors and, as expected, impacts cooperativity of the system. MD simulations also predicted that the M8R mutation shifts the equilibrium position of tropomyosin toward its B-state on actin. The Markov model uses the equilibrium constant K_BC_ to encode the ratio of the transition rate towards the C-state to the transition rate towards the B-state (in the absence of TnI inhibition). Thus, the change to a more B-state-like position was encoded as a decrease in the K_BC_ parameter.

To find the best fit of the mutant velocity-pCa curve to the experimental IVMA data, a range of ten values each for γ and K_BC_ were selected and run in combination to perform a grid search (Figure 1B). The sum of squared errors (SSE) with respect to the experimental data for the percent change in maximum velocity (V_max_) and absolute change in pCa_50_ was calculated for each parameter combination (Figure 1B). This grid of simulations enables direct visualization of the error landscape and reveals a single minimum SSE well. This point lies approximately at a parameter combination equivalent to decreasing γ by 50% (from 70 to 35) and simultaneously decreasing K_BC_ by 26% (from 2.3 to 1.7). These changes reproduced the rightward shift in pCa_50_ and the drop in V_max_ that matched the experimental means to well within their ranges of uncertainty (Figure 1C). This combination also qualitatively matched the drop in n_H_ that was observed in the IVMA curves.

### 3.2 Twitch simulations

Once appropriate percent changes in γ and K_BC_ were found that reflected the steady-state M8R experimental data, they were applied to the model to predict how M8R might affect isometric cardiac muscle contractions. Because the IVMA used 100% M8R mutant tropomyosin, the parameter changes determined from the IVMA fit were assumed to reflect 100% expression of M8R. Genotypes in sarcomeric forms of DCM are typically heterozygous such that the disease-relevant expression of *TPM1* M8R would be approximately 50%. We therefore simulated intermediate expression levels of *TPM1* M8R, assuming a linear interpolation between WT and M8R mutant model parameters in proportion to the specified expression ratio.

**FIGURE 2:**
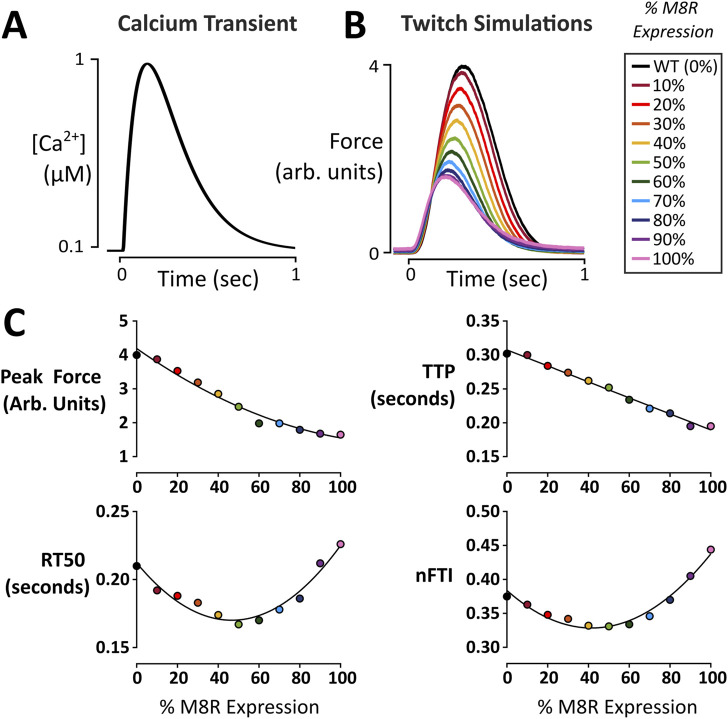
Twitch force simulations as a function of M8R expression. **(A)** Ca^2+^ transient used to produce twitch simulations; **(B)** Force output of model simulations over a range of M8R expression; **(C)** Twitch characteristics at each expression level.

A parameter set was selected to reproduce twitch kinetics of a representative human engineered heart tissue (EHT) (Table 1) to serve as a baseline (WT or 0% expression of M8R). The change for 100% M8R expression was applied to γ and K_BC_ and was then decreased by 10% per run down through 10% expression. Throughout the range of expression, there were minor changes to the Ca^2+^ transient, but overall, they were quite similar despite the change in Ca^2+^ sensitivity seen in the M8R steady-state simulations (Figure 2B). For each twitch, the peak force, time to peak force (TTP), time from peak force to 50% relaxation (RT50), and normalized force-time integral (nFTI, area under the twitch force curve divided by peak force) were calculated. It was found that with increasing percentage of M8R expression, the size of the twitches decreased, as expected for a DCM-linked mutation (Hasenfuss et al., 1992; Mijailovich et al., 2021; Powers et al., 2020) (Figure 2C). The peak force and TTP decreased proportionally through the range of expression. However, the RT50 and nFTI had a biphasic response, first falling and then rising as a function of increasing M8R expression (Figure 2D).

### 3.3 Engineered heart tissue

**FIGURE 3:**
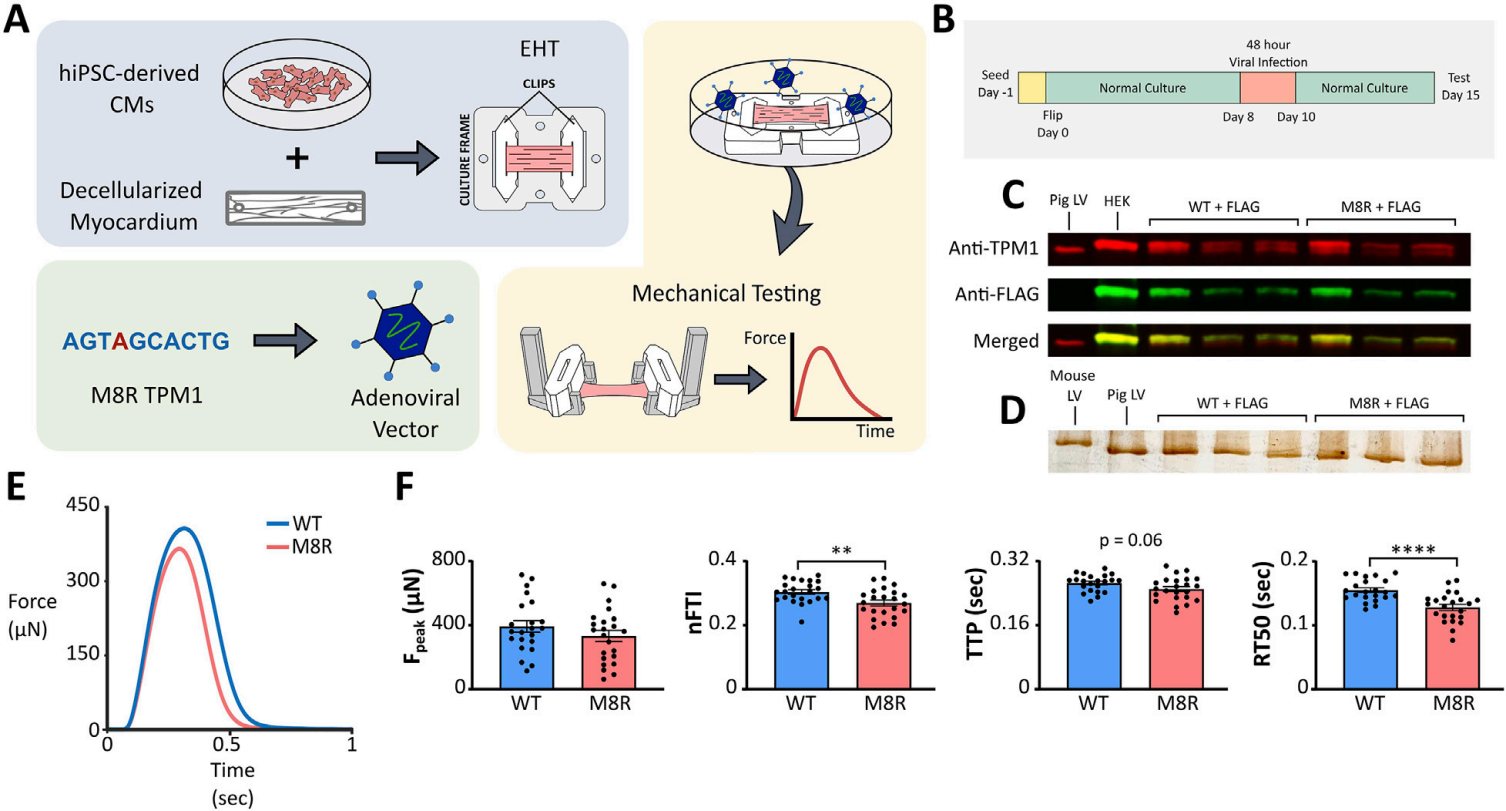
**(A)** Schematic of engineered tissue and adenovirus transduction process; **(B)** EHT timeline; **(C)** Western blot for tropomyosin and FLAG expression. Controls in first two lanes (pig left ventricle and HEK cells transduced with WT *TPM1* + FLAG) compared to EHTs transduced with WT *TPM1* + FLAG in next three lanes and *TPM1* M8R + FLAG in final three lanes; **(D)** Silver stain for myosin isoforms. Controls in first two lanes (mouse ventricle and pig left ventricle) compared to EHTs transduced with WT *TPM1* + FLAG in next three lanes and *TPM1* M8R + FLAG in final three lanes; **(E)** Representative twitch force records obtained in EHTs transduced with WT *TPM1+*FLAG (WT) or *TPM1* M8R + FLAG (M8R), selected for exhibiting peak force levels near the mean values obtained in the respective groups; **(F)** Comparison of twitch characteristics in WT vs. M8R EHTs: Peak force (F_peak_), normalized force-time integral (nFTI), time from stimulus to peak force (TTP), and time from peak force to 50% relaxation (RT50).

We next sought independent evidence that the complex model-predicted effects of M8R expression on isometric twitches were reasonable. EHTs were produced from human induced pluripotent stem cell-derived cardiomyocytes and transduced with either *TPM1* M8R or WT *TPM1* using adenoviral vectors (Figure 3A). Expression of FLAG-tagged protein was confirmed with Western blot (Figure 3C). The Western blot compared pig left ventricle expressing native *TPM1* and HEK cells transduced with *TPM1*+FLAG to cardiac tissues that had been transduced with either WT *TPM1* + FLAG or M8R *TPM1* + FLAG. *TPM1* with FLAG was estimated to replace 48%–60% of *TPM1* in the transduced tissues. Seven days after viral treatment, EHTs were removed from culture and subjected to isometric force measurements (Figure 3B). Representative traces can be seen in Figure 3C. EHTs expressing M8R showed significant decreases in RT50 and nFTI of 17.5% and 11.4% respectively. The TTP decrease of 5.6% was of marginal significance (*p* = 0.06), and there was a trend towards decreased peak force, with a 15.3% drop between means.

After testing, tissues were flash frozen for myosin isoform analysis. A silver stain was run comparing cardiac tissues that had been transduced with either WT *TPM1* + FLAG or M8R *TPM1* + FLAG to samples of mouse ventricle and pig left ventricle (Figure 3D). Mouse ventricle is primarily composed of α-myosin, while pig left ventricle is primarily β-myosin. In agreement with previous reports (Ng et al., 2021), our EHTs express essentially 100% β myosin, aligning with the pig left ventricular samples. This was unchanged by transduction with M8R *TPM1* + FLAG, indicating that the alterations in twitch kinetics are not due to myosin isoform shift.

### 3.4 Comparison of model prediction and *in vitro* data

**FIGURE 4:**
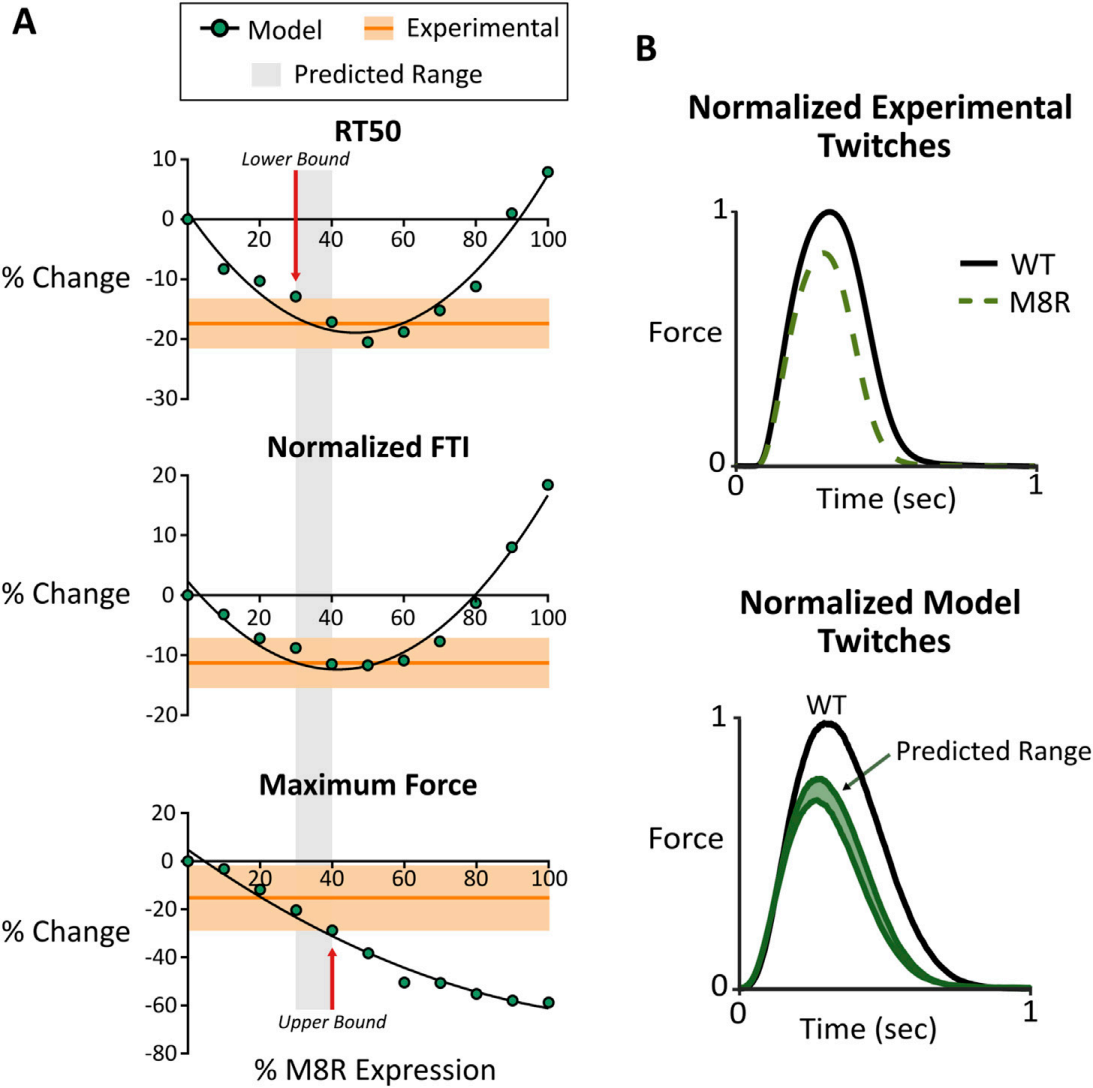
**(A)** Comparison between computer model output and engineered tissue data for RT50, normalized FTI, and maximum force. Modeling data is shown with changing expression of M8R. Experimental data is shown with the mean in dark orange and the range of SEM in light orange; **(B)** normalized twitch plots for experimental tissue data and model output showing comparison of predicated range and actual phenotype outputs.

Ideally, a quantitative comparison between modeled and measured tropomyosin M8R effects on isometric twitch force would be accomplished by measuring mutant protein and applying that expression level to the model. Although Western blot data confirm robust expression of FLAG-tagged mutant tropomyosin (Figure 3C), they do not provide an explicit expression level, nor do we know the degree to which the mutant tropomyosin is incorporated in the muscle lattice. Therefore, evaluation of model predictions required a more indirect approach. The model predicts that twitch properties including RT50, nFTI, and peak force vary distinctly as a function of M8R expression (Figure 4A). We reasoned that the model and experiments could be deemed quantitatively consistent with each other if an expression level could be found for which simulated M8R-mediated percent change in peak force, RT50, and nFTI matched experiments to within the observed standard error.

The comparison between simulated and measured changes in twitch properties is seen in Figure 4A. These plots suggest that M8R expression must be at least 30%, because any expression level lower than this will cause the model to predict too small a drop in RT50. Meanwhile, the upper bound of expression appears at 40%, above which the drop in peak twitch force would exceed the experimentally observed range. Overall, a predicted range of 30%–40% adenoviral-expressed mutant tropomyosin causes all three twitch metrics to agree between model and experiment.

Looking at the predicted range of the model on a normalized simulated twitch and comparing it with a normalized representative trace from the engineered tissue data, there are clear similarities in phenotype (Figure 4B). This suggests that the molecular-scale pathologies ascribed to the M8R mutation are sufficient to explain the measured phenotype in a physiologically relevant system with a mixture of WT and mutant tropomyosin.

### 3.5 Independent effects of changes

**FIGURE 5:**
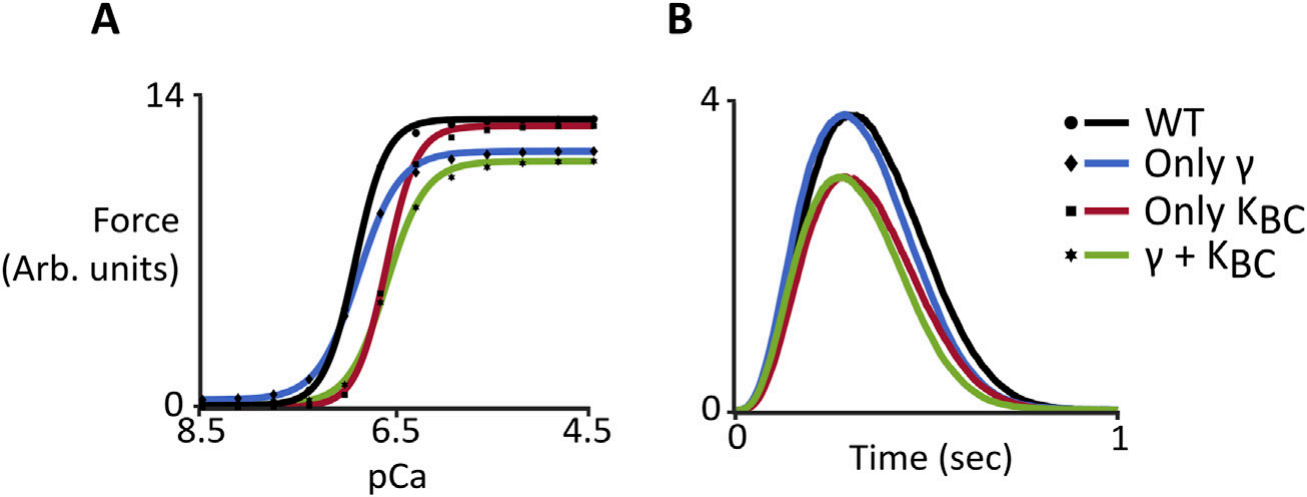
Computer simulations comparing wild-type behavior and changes to γ and K_BC_ both independently and together. **(A)** Steady-state force-pCa curves. Symbols show model output and solid lines show the fit of the Hill Equation; **(B)** Dynamic behavior of simulated twitches.

While there is clear evidence for both decreased stiffness and favoring of the B-state caused by the M8R mutation, the degree to which each change contributes to physiological behavior is not obvious. The computational representation of M8R molecular, structural, and mechanical effects as model parameters gives the ability to independently examine their impact on phenotype. While increasing M8R expression decreases both γ and K_BC_ in this model, we wanted to see the effect of changing only one or the other compared to the effects of both in combination. For steady-state simulations (Figure 5A), changing only γ produced the observed drops in force and n_H_, but only created a mild rightward shift in pCa_50._ On the other hand, decreasing K_BC_ alone, resulted in only the shift in Ca^2+^ sensitivity. The additive effect of the two changes is necessary to capture characteristics of the IVMA data. Meanwhile the twitch simulations (Figure 5B) showed the desired shift in kinetics when γ was changed but only a mild drop in force. The much larger contribution to the mutant phenotype of M8R came from the decrease in K_BC_, which dropped peak force to levels on par with the full 100% expression of both parameters.

## 4 Discussion

The objective of this study was to use a multiscale approach that couples *in silico* predictions with *in vitro* experimental data to give insights on the genotype-phenotype relationship for the M8R mutation to human α-tropomyosin. This mutation is linked to a DCM phenotype and simulations from previous work hypothesized that this occurs due to decreased effective chain stiffness in the overlap region of tropomyosin as well as a shift to a more B-state-like position. However, the critically important link between these molecular effects and the contractile phenotype is not known. Our Markov model of the cardiac thin filament was used to recreate the IVMA steady-state curves as a starting point for validation of these mechanistic insights. IVMA with filaments reconstituted with M8R tropomyosin showed a rightward shift in Ca^2+^ sensitivity, a drop in maximum velocity, and a decrease in n_H_. The computer model was able to capture a quantitative change in Ca^2+^ sensitivity and maximum velocity, as well as a qualitative drop in n_H_. Significantly, the drop in effective tropomyosin stiffness (γ) predicted by fitting IVMA data corresponds closely with the drop in persistence length through the tropomyosin-tropomyosin overlap region predicted by MD simulations (a 50% decrease in both cases).

Running twitch simulations over a range of M8R expression levels demonstrated that increased expression decreased force in the twitches proportionally. However, the relaxation and normalized force-time integral for the twitches changed in a biphasic manner. That is, introducing M8R expression initially decreased both measures but they both increased to higher levels than WT above ∼50% expression of mutant tropomyosin. In the model, this complex response is caused by mutation-induced alterations in cooperativity between regulatory units (γ). The coupling of adjacent regulatory units causes two related but distinct forms of Ca^2+^-based cooperativity (Figure 2E): At low levels of Ca^2+^ activation, inactive regulatory units can pull neighbors into or maintain them in an inactive state (cooperative *inhibition*). At higher levels of Ca^2+^ activation, an active regulatory unit can pull inactive neighboring units into or maintain them in an active state (cooperative *activation*). A decrease in γ initially leads to a shorter relaxation time because activated tropomyosin molecules are less capable of keeping their neighbors open as Ca^2+^ concentration is falling; thus leading to an overall decreased twitch duration. However, further drop in γ also erodes the overall peak twitch activation level (due to loss of cooperative activation). At these low levels of activation, and with dropping γ, the inability of neighboring tropomyosin molecules to inactivate adjacent regulatory units dominates relaxation, and RT50 begins to increase. This same phenomenon explains the biphasic character of normalized force-time integral as a function of M8R expression.

To validate the model’s prediction of isometric twitch behavior, we produced EHTs that expressed *TPM1* M8R using adenoviral vectors. Comparison of WT- and M8R-expressing EHTs showed that the mutant tissues had a trend towards decreased peak force and significant decreases in relaxation time and normalized area under the twitch. This contractile dysfunction is unsurprising given that the overarching DCM phenotype in patients is severe heart failure (Weintraub et al., 2017). Computer-simulated twitches also predicted a hypocontractile phenotype, showing general agreement between both model systems. Specific agreement between measured and simulated twitches was not directly performed since it is difficult to determine the ratio of native tropomyosin to M8R tropomyosin in the tissues. However, an indirect analysis was possible using the model’s output to ascertain a range of expressions that would produce close simultaneous agreement of multiple twitch parameters. An overlay of the range of experimental data and the output from the model showed a small range of 30%–40% replacement where the two coincided for peak force, RT50, and nFTI. This is a realistic amount of expression expected from adenoviral expression and the overlap of data in all three measurements suggests that the model’s prediction is reasonable. In addition, it is an expression level that approaches the 50% expression we would expect to see clinically for dominant mutation since most cardiomyopathy patients are heterozygous for the disease. As such, the data produced by the M8R EHTs may be comparable to an expected clinical phenotype.

One extra benefit of using the Markov model compared to an *in vitro* system alone is the ability to separate the contribution of the mechanistic changes. While the MD simulations indicated both the decrease in chain stiffness and shift of position of tropomyosin occurring in M8R, there was no way to parse out the magnitude of change occurring due to each these disruptions. The computer model provided an opportunity to apply the stiffness change alone and the shift to a more B-state equilibrium alone as well as in combination. This highlighted the influence of γ and K_BC_ individually on the functional changes for steady-state and dynamic force production. It was seen that, while both parameters are required to produce a combined effect to match experimental data in the force-pCa curves, the isometric twitch data suggests that the change to K_BC_ has the dominating effect on controlling the DCM phenotype observed in M8R. This indicates that the position of tropomyosin on actin may be more important than changes to flexibility in contributing to cardiac twitch dysfunction.

One limitation noted for this study is that a fixed Ca^2+^ transient was used in the twitch simulations to match force predictions at various ratios of mutant protein expression. This does not account for mutation-based effects on Ca^2+^ sensitivity, which would alter intracellular Ca^2+^ buffering by troponin C and ultimately modify the Ca^2+^ transient. Predicting these impacts in a model would require a depiction of cardiomyocyte electrophysiology and Ca^2+^ handling to be coupled with the stochastic Markov myofilament activation model. Such a model would enable more confident predictions of the level of virally expressed mutant tropomyosin in EHTs but its implementation is not trivial and lies outside the scope of this study.

The data collected thus far by this and previous studies are consistent with other DCM mutations through 1) changes to the equilibrium position of tropomyosin on actin found in the MD simulations to resemble a blocked state position when in the closed state, 2) decreased Ca^2+^ sensitivity, cooperativity, and maximum force as seen in the IVMA data, and 3) our engineered tissue model in which we see decreased relaxation time and normalized twitch size. We believe that the linkage analysis results combined with the *in silico* and *in vitro* work in this study supports a pathogenic designation for *TPM1* M8R.

*TPM1* is only one of the many sarcomeric genes that have been linked to DCM; mutations in proteins such as titin, TnC, TnI, TnT, myosin, and actin have also been implicated (Carballo et al., 2009; Huang et al., 2015; Landim-Vieira et al., 2020; Song et al., 2010; Du et al., 2007; Sun et al., 2012; Hinson et al., 2015; Hershberger et al., 2013; Gomes and Potter, 2004; Tadros et al., 2020). However, mutations in TnT, TnI, and TnC can also cause hypertrophic cardiomyopathy, and one particular mutation in TnI has been shown to cause both restrictive or hypertrophic cardiomyopathy (Gomes and Potter, 2004). Molecular mechanisms of these sarcomeric DCM mutations vary, but can include defects in force generation or transmission, impaired sarcomereogenesis, changes in calcium sensitivity, energy deficits, or alterations in troponin subunit interactions (Johnston et al., 2019; Fatkin and Graham, 2002; Yotti et al., 2019). Continued efforts are being made by our group and others to analyze the relationship between sarcomeric mutations and their consequences that lead to a DCM phenotype, given that guidelines for genetic evaluation of cardiomyopathy patients continue to evolve and many mutations cannot be unambiguously linked to a clinical phenotype (Chang and Potter, 2005; Teo et al., 2015; Deranek et al., 2019). We acknowledge that DCM-causing mutations are not limited to sarcomeric proteins, and include desmosomal, nuclear, cytoplasmic, membrane-associated, and mitochondrial proteins (McNally and Mestroni, 2017). Finally, while mutations to tropomyosin can lead to DCM, they can also result in a hypertrophic cardiomyopathy (HCM) phenotype (Jääskeläinen et al., 2013; García-Castro et al., 2009; Karibe et al., 2001; Jongbloed et al., 2003; Van Driest et al., 2002; McNally et al., 2013). Given immense heterogeneity in the genetics and pathogenesis of cardiomyopathy, multiscale modeling techniques may provide an effective means of relating variants of unknown significance to their predicted phenotype.

## Data Availability

The raw data and computer simulation code supporting the conclusions of this article will be made available by the authors, without undue reservation.

## Abbreviations

DCM, dilated cardiomyopathy; TPM1, human alpha-tropomyosin gene; IVMA, in vitro motility assay; MD, molecular dynamics; WT, wild-type.
