# Supplementary material for: In silico and in vitro models reveal the molecular mechanisms of hypocontractility caused by TPM1 M8R
Source: Front Physiol. 2024 Aug 30;15:1452509. doi: 10.3389/fphys.2024.1452509 (PMC11392859; doi:10.3389/fphys.2024.1452509)
Supplement: Supplementary file 1 [file DataSheet1.PDF]

Supplemental Material

In Silico and In Vitro Models Reveal the Molecular Mechanisms of Hypocontractility Caused by *TPM1* M8R

A.

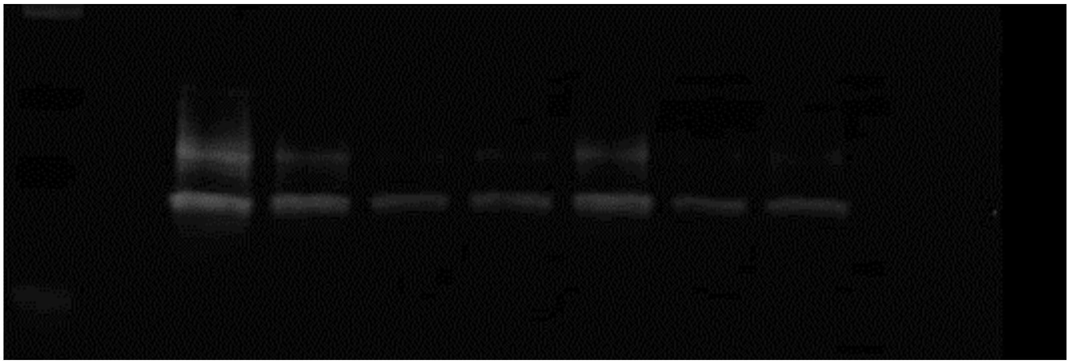

B.

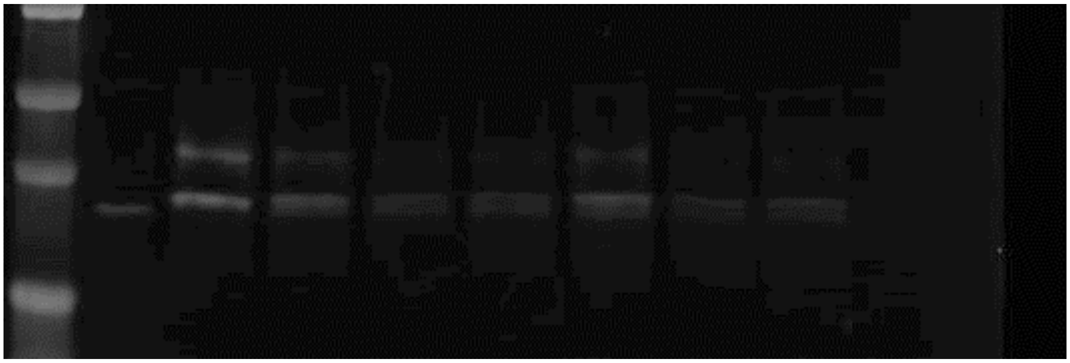

Supplemental Figure 1: Western blot scanned .tiff files used in Figure 3. A) Anti-FLAG imaged in 800 channel; B) Anti-TPM1 imaged in 700 channel.

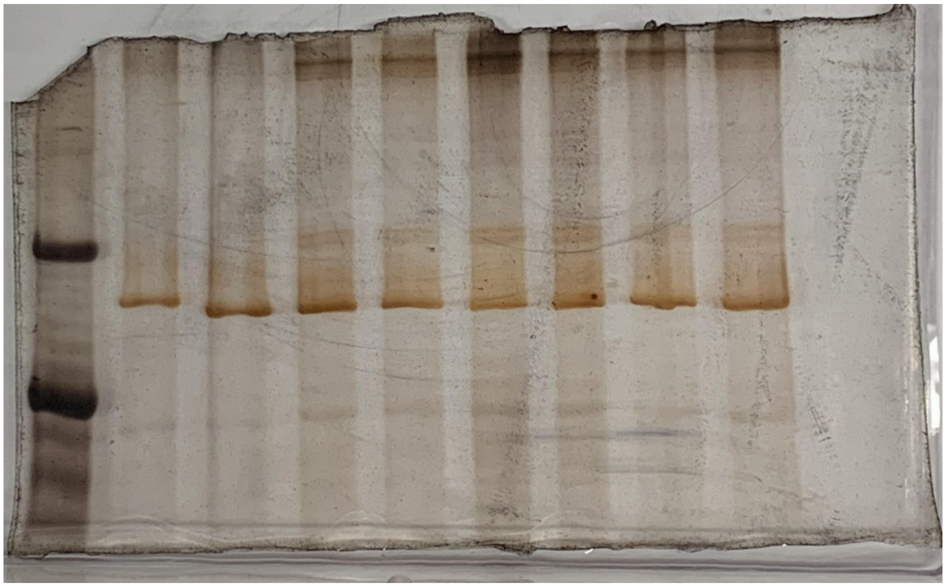

Supplemental Figure 2: Full silver stain used in Figure 3.

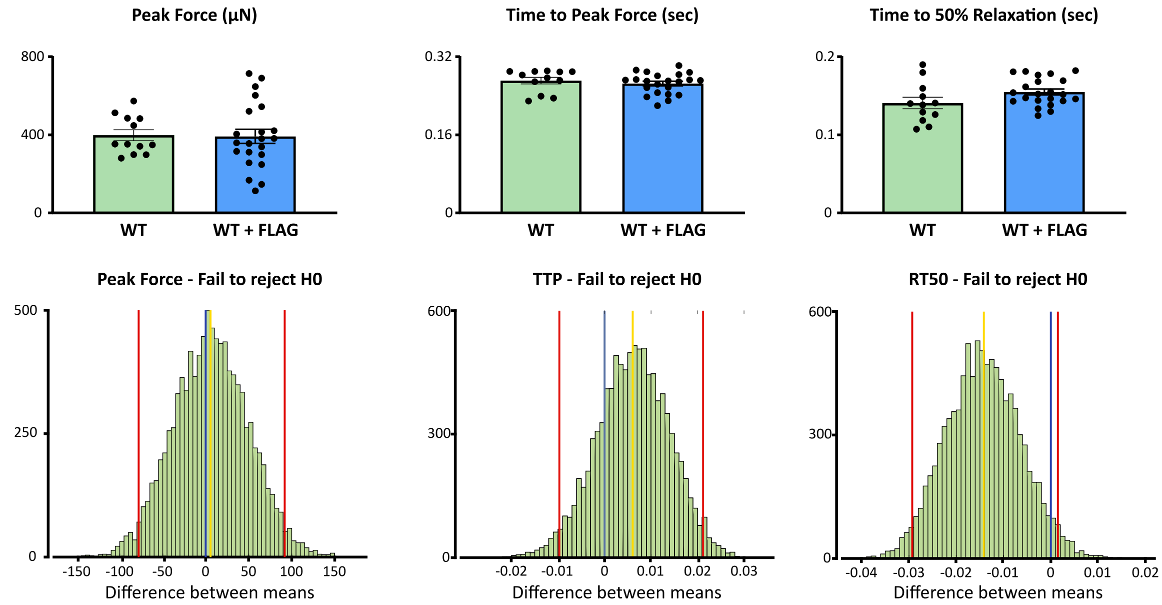

Supplemental Figure 3. Bootstrap analysis of tissues transduced with WT TPM1 vs WT TPM1 + FLAG.
